# Association between immune-related adverse events and the efficacy of PD-1 inhibitors in advanced esophageal cancer

**DOI:** 10.3389/fimmu.2022.931429

**Published:** 2022-09-28

**Authors:** Wenru Qin, Linlin Yang, Bingjie Fan, Bing Zou, Yanan Duan, Butuo Li, Linlin Wang

**Affiliations:** ^1^ Department of Radiation Oncology, Shandong Cancer Hospital and Institute, Shandong First Medical University and Shandong Academy of Medical Sciences, Jinan, China; ^2^ Department of Oncology, Shandong First Medical University, Jinan, China

**Keywords:** esophageal cancer, immune-related adverse events, PD-1 inhibitors, immune checkpoint inhibitors, prognostic marker

## Abstract

**Introduction:**

Recent developments in immune checkpoint inhibitors (ICIs) have improved the treatment outcomes of esophageal cancer (EC); however, it may initiate immune-related adverse events (irAEs) in some patients. The ICIs’ therapeutic efficacy is associated with irAEs in patients with non-small cell lung cancer or renal cell carcinoma, although this association is unknown in EC. The purpose of this study was to explore the association between irAEs and the efficacy of programmed death 1 (PD-1) inhibitors in EC patients.

**Patients and methods:**

This study included patients with advanced EC treated with PD-1 inhibitors. The patients were divided into two groups according to the occurrence of irAEs. Afterward, the efficacy was compared between the irAE-negative and irAE-positive groups, and we analyzed the predictive factors of irAEs and survival.

**Results:**

Overall, 295 patients were included in this study. Baseline characteristics were balanced in the irAE-negative and irAE-positive groups. In total, 143 (48.47%) patients experienced irAEs. The most frequent irAEs were anemia (49, 16.61%), hyperthyroidism (45, 15.25%), and pneumonitis (44, 14.92%). In total, 33 (11.19%) patients had grade ≥ 3 irAEs and pneumonitis have 15 (5.08%). No grade 5 adverse events were observed. A total of 52 (17.63%) and 91 (30.85%) patients had single and multiple irAEs, respectively. Compared with patients without irAEs, those with irAEs had significantly higher objective response rate (ORR) (37.76% vs. 25.00%, p = 0.018) and disease control rate (DCR) (92.31% vs. 83.55%, p = 0.022). Univariate Cox analyses indicated the significant association between irAEs and improved median progression-free survival (PFS) (10.27 vs. 6.2 months, p < 0.001) and overall survival (OS) (15.4 vs. 9.2 months, p < 0.001). In multivariate analyses, irAEs were independently associated with longer PFS (p = 0.011) and OS (p = 0.002). Moreover, multivariate analysis revealed that cycles > 8, radiation, as well as antiangiogenic therapy were strongly associated with irAEs development (p < 0.001, p = 0.002, and p = 0.025, respectively).

**Conclusion:**

In advanced EC, patients with irAEs showed markedly better efficacy in ORR, DCR, PFS, and OS compared with patients without irAEs.

## Introduction

Esophageal cancer (EC) ranks seventh in the incidence of cancer and the sixth most frequent cause of cancer-related death worldwide (1). However, conventional radiotherapy and chemotherapy have limited efficacy and cause serious adverse effects for EC patients. Recently, immune checkpoint inhibitors (ICIs) have become an essential and promising therapy for advanced EC (2). Programmed death 1 (PD-1) and programmed death-ligand 1 (PD-L1) are immune checkpoints, whose inhibitors have been found to trigger T lymphocytes, inhibit the growth of cancer cells, and improve survival in cancer patients (3, 4). Until recently, nivolumab, pembrolizumab, and camrelizumab plus chemotherapy, which have resulted in a more prolonged overall survival (OS) and progression-free survival (PFS) compared to cytotoxic chemotherapy, are recommended as first-line treatment for advanced EC patients (5–8).

However, it has to be noticed that ICIs can cause immune-related adverse events (irAEs), which may occur in any organ system and may be permanent or even life-threatening. IrAEs might impair quality of life or even lead to death (9). The incidence rate of irAEs of any grade has been reported to be 66% with PD-1/L1 inhibitors, and combination therapy can increase the risk of irAEs in patients with multiple solid tumor types (10–13). Although the mechanism of irAEs is unclear, a potential mechanism might be that ICIs enhance systemic T-cell activity resulting in the loss of immune tolerance in individual organs, which causes irAEs (14). Some retrospective studies have claimed that the occurrence of irAEs is associated with better treatment response or prognosis, such as objective response rate (ORR), disease control rate (DCR), PFS, and OS, in renal cell carcinoma and non-small cell lung cancer (NSCLC) (15–19). However, currently, reliable data regarding the relationship between irAEs and prognosis in patients with advanced EC treated with PD-1 inhibitors are insufficient.

In this study, we aimed to investigate the potential association between irAEs and outcomes of PD-1 inhibitors and identify factors related to the outcomes of PD-1 inhibitors treatment in patients with advanced EC.

## Methods

### Patients

Patients with histologically confirmed EC who had been treated with ICIs therapy at least two doses between January 2018 and August 2021 at Shandong Cancer Hospital and Institute, Shandong First Medical University were included in this study. Patients who had previously received ICIs were excluded. We reviewed the medical records and the following patient characteristics prior to initiation of ICIs treatment: age, sex, Eastern Cooperative Oncology Group Performance Status (ECOG PS), stage, histology, history of surgery, metastatic sites, immunotherapy line, and lactate dehydrogenase (LDH) level. The PS at the initiation of ICIs therapy was evaluated by the ECOG PS scale. The disease stage was evaluated on the basis of the American Joint Committee on Cancer VIII staging system.

Clinical assessments were performed by the Response Evaluation Criteria in Solid Tumors version 1.1 criteria at baseline and every 2-3 courses (every two months). The best overall response was defined as the best response achieved after the initiation of PD-1 inhibitors. Data regarding irAEs were collected from clinical notes, hospitalization records, and laboratory values. All irAEs were graded by the senior doctors according to the Common Terminology Criteria for Adverse Events version 5.0. Multiple irAEs were defined as irAEs of ≥ 2.

All patients were divided into an irAE-positive group (with irAEs) and an irAE-negative group (without irAEs) based on the occurrence of irAEs. Differences in efficacy were analyzed between the irAE-positive and irAE-negative groups.

### Statistical analysis

Categorical data were analyzed based on the chi-squared test, and Student’s t-test was performed to analyze quantitative data. Survival data were evaluated with both Kaplan-Meier and log-rank tests. Logistic regression analyses were used to determine whether data were associated with irAEs. Univariate and multivariate comparisons of PFS and OS were performed using Cox proportional hazards regression models. A two-tailed p < 0.05 was considered statistically significant. Statistical analyses were performed with IBM SPSS 26.0.

## Results

### Patient characteristics

This study included 295 patients. The median age was 60 (range, 36–84) years, the majority were male (259, 87.8%), and 210 (71.2%) patients received at least one prior systemic treatment. 23 were treated with immunotherapy alone, 95 with immunotherapy combined with chemotherapy, 148 with immunotherapy combined with radiotherapy, and 29 with immunotherapy combined with antiangiogenic therapy.

There were no significant differences in baseline characteristics between patients with and without irAEs (**Supplementary Table 1**).

### Incidence of immune-related adverse events

In total, 143 (48.47%) patients experienced irAEs. The most frequent irAEs were anemia (49, 16.61%), hyperthyroidism (45, 15.25%), and pneumonitis (44, 14.92%). In total, 33 (11.19%) patients experienced ≥ 3 grade irAEs, with the most frequent being pneumonitis (15, 5.08%). It was not observed that grade 5 adverse events related to immunotherapy. A total of 52 (17.63%) and 91 (30.85%) patients had single and multiple irAEs, respectively. Twenty-three of the patients who experienced irAEs were treated with glucocorticoid for serious irAEs, and 10 patients with endocrine irAEs required hormonal replacement therapy. The details of irAEs are described in **Table 1**.

**Table 1:** Immune-related adverse events according to category and grade.

Category	Total N (%)	Grade 1-2 N (%)	Grade 3-4 N (%)
Any	143 (48.47)	110 (37.29)	33 (11.19)
Anemia	49 (16.61)	44 (14.92)	5 (1.69)
Hyper/hypothyroidism	45 (15.25)	43 (14.58)	2 (0.68)
Pneumonitis	43 (14.58)	28 (9.49)	15 (5.08)
Cardiovascular toxicities	35 (11.86)	32 (10.85)	3 (1.02)
Fatigue	23 (7.80)	22 (7.46)	1 (0.34)
Thrombocytopenia	22 (7.46)	20 (6.80)	2 (0.68)
Fever	17 (5.76)	17 (5.76)	0 (0)
Elevated transaminase	16 (5.42)	13 (4.41)	3 (1.02)
Anorexia	15 (5.08)	1 (0.34)	0 (0)
Reactive cutaneous capillary endothelial proliferation	13 (4.41)	12 (4.07)	1 (0.34)
Nausea/vomiting	10 (3.39)	10 (3.39)	0 (0)
Rash	5 (1.69)	4 (1.36)	1 (0.34)
Pruritus	5 (1.69)	5 (1.69)	0 (0)
Arthralgia/Myalgia	4 (1.36)	4 (1.36)	0 (0)
Diarrhea/colitis	1 (0.34)	1 (0.34)	0 (0)

### Association between irAEs and the efficacy of PD-1 inhibitors

**Figure 1:**
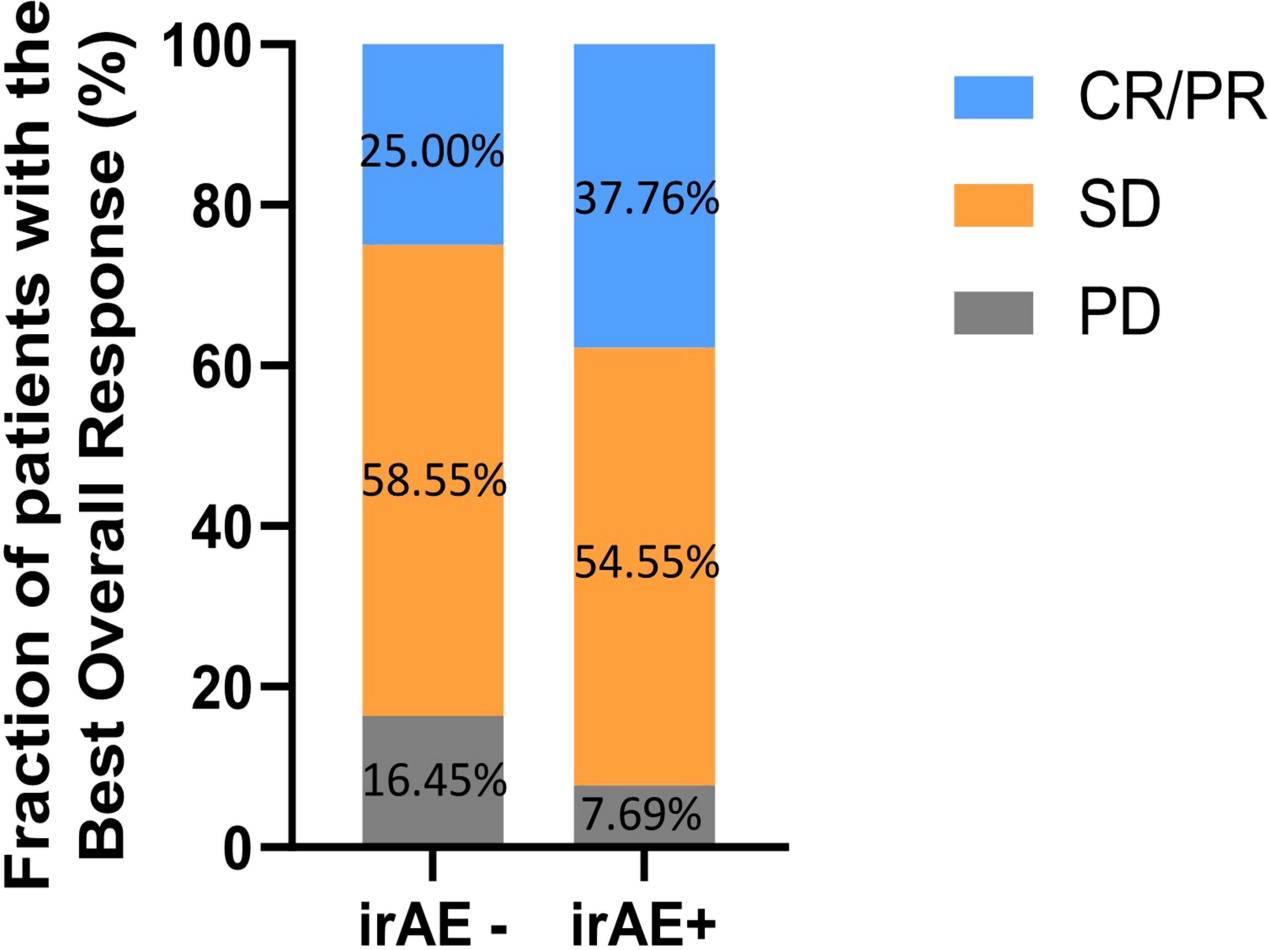
The fraction of patients with the best overall response in patients with or without irAEs. CR, complete response; PR, partial response; SD, stable disease; PD, progressive disease; irAEs, immune-related adverse events.

Patients who presented with irAEs had better ORR (37.76% vs. 25.00%, p = 0.018) and DCR (92.31% vs. 83.55%, p = 0.022) than those without irAEs, as shown in **Figure 1** and **Table 2**.

**Table 2:** Best overall response during PD-1 inhibitors.

	All patients	irAE - group	irAE + group	p value
Total	295	152	143	
CR/PR	92	38	54	
SD	167	89	78	
PD	36	25	11	
ORR, %	31.19	25.00	37.76	0.018*
DCR, %	87.80	83.55	92.31	0.022*

CR, complete response; PR, partial response; SD, stable disease; PD, progressive disease; ORR, objective response rate; DCR, disease control rate; irAEs, immune-related adverse events.

*p<0.05.

**Figure 2:**
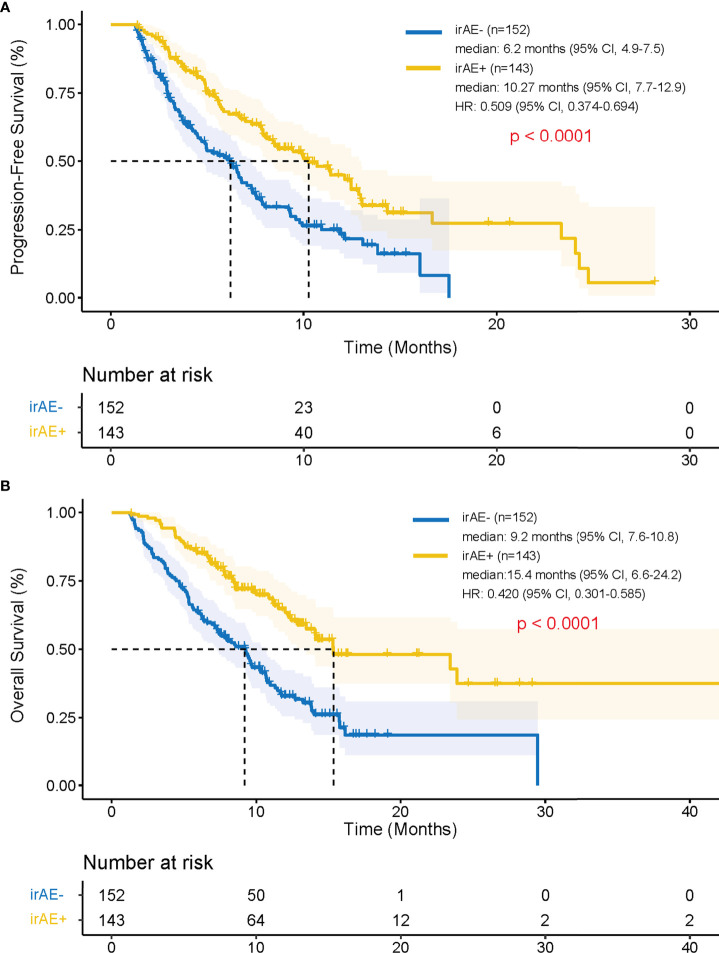
Progression-free survival and overall survival after the treatment of PD-1 inhibitors depending on the development of irAEs using Kaplan-Meier method. Kaplan–Meier curves for **(A)** progression-free survival and **(B)** overall survival in patients with or without irAE. irAEs, immune-related adverse events; HR, hazard ratio; CI, confidence interval.

Patients in irAE-positive group had higher median PFS compared with patients in the irAE-negative group (10.27 months vs. 6.2 months; hazard ratio [HR], 0.509; 95% confidence interval [CI], 0.374–0.694; p < 0.001) (**Figure 2A**). Patients in the irAE-positive group had higher median OS compared with those in the irAE-negative group (15.4 months vs. 9.2 months; HR, 0.420; 95% CI, 0.301–0.585; p < 0.001) (**Figure 2B**).

Interestingly, even in patients who received ICIs for ≤ 8 cycles (n = 221), the median PFS and OS were significantly longer in the irAE-positive group (n = 92) than in the irAE-negative group (n = 129) (PFS: 5.7 months vs. 4.6 months; HR, 0.682; 95% CI, 0.488–0.953; p = 0.024; OS: 11.2 months vs. 7.1 months; HR, 0.585; 95% CI, 0.413–0.829; p = 0.002).

**Figure 3:**
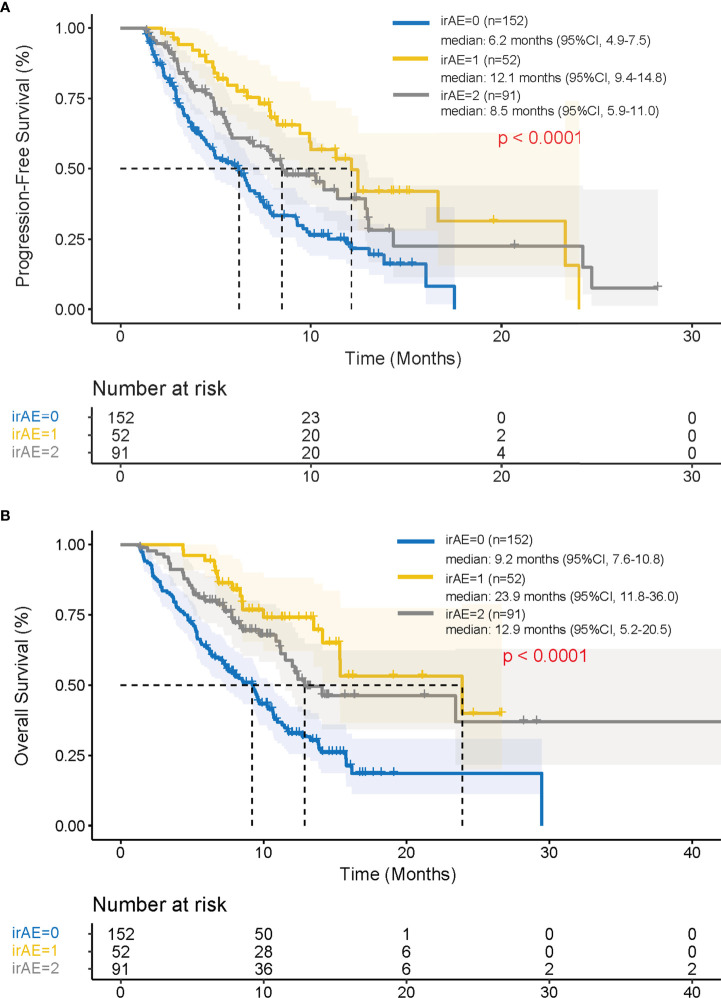
Progression-free survival and overall survival after the initiation of PD-1 inhibitors depending on the number of irAEs using Kaplan-Meier method. Kaplan–Meier curves for **(A)** progression-free survival and **(B)** overall survival in patients with ≥2 irAEs compared with those with one irAE and none irAEs. irAEs, immune-related adverse events; CI, confidence interval.

When analyzing survival outcomes based on the number of irAEs, patients who presented with a single irAE (n = 52) had a significantly longer PFS and OS compared to those with ≥ 2 irAEs (n = 91) or who did not experience irAEs (n = 152) (PFS: 12.1 vs. 8.5 vs. 6.2 months, p < 0.001; OS: 23.9 vs. 12.9 vs. 9.2 months, p < 0.001) (**Figures 3A, B**).

Among the various irAEs, no significant association between pneumonia or skin et al. irAEs and survival was observed in our study.

### Univariate and multivariate cox analyses of PFS and OS

Univariate analysis revealed that ECOG PS ≥ 2, number of organs with metastases ≥ 2, cycles ≤ 8, LDH level > the upper limit of normal (ULN), and without irAEs were significantly associated with shorter PFS. Multivariate analysis demonstrated that ECOG PS ≥ 2, cycles ≤ 8, LDH level > the ULN, and no irAEs were independent factors for worse PFS (**Table 3**).

**Table 3:** Results of univariate and multivariate analyses showing factors affecting the progression-free survival.

Variables	Univariate analysis		Multivariate analysis	
	HR for PFS (95% CI)	p value	HR for PFS (95% CI)	p value
**ECOG PS**				
<2	1.750 (1.296-2.362)	<0.001*	1.607 (1.170-2.206)	0.003*
≥2				
**Number of organs with Metastases**				
<2	1.408 (1.028-1.928)	0.033*	1.366 (0.975-1.913)	0.070
≥2				
**Immunotherapy line**				
1st	1.270 (0.911-1.771)	0.159		
≥2nd				
**Cycles**				
≤8	0.177 (0.115-0.273)	<0.001*	0.196 (0.125-0.307)	<0.001*
>8				
**Radiation**				
No	0.720 (0.514-1.007)	0.055		
Yes				
**LDH level**				
≤ULN	1.440 (1.006-2.063)	0.046*	1.416 (0.965-2.079)	0.076
>ULN				
**Group**				
irAE(-)	0.509 (0.374-0.694)	<0.001*	0.655 (0.474-0.907)	0.011*
irAE(+)				

ECOG PS, Eastern Cooperative Oncology Group Performance Status; HR, hazard ratio; CI, confidence interval; LDH, lactate dehydrogenase; ULN, upper limit of normal; irAEs, immune-related adverse events; PFS, progression-free survival. *p<0.05.

Univariate analysis showed that ECOG PS ≥ 2, immunotherapy line ≥ 2nd, cycles ≤ 8, no radiation, LDH > the ULN, and no irAEs were associated with poor OS. Multivariate analysis of OS showed that ECOG PS ≥ 2, immunotherapy line ≥ 2nd, cycles ≤ 8, no radiation, and no irAEs were independent poor prognostic factors (**Table 4**).

**Table 4:** Results of univariate and multivariate analyses showing factors affecting the overall survival.

Variables	Univariate analysis		Multivariate analysis	
	HR for OS (95% CI)	p value	HR for OS (95% CI)	p value
**ECOG PS**				
<2	1.703 (1.243-2.335)	0.001*	1.470 (1.056-2.046)	0.022*
≥2				
**Number of organs with Metastases**				
<2	1.034 (0.739-1.448)	0.845		
≥2				
**Immunotherapy line**				
1st	2.052 (1.390-3.030)	<0.001*	1.755 (1.179-2.612)	0.006*
≥2nd				
**Cycles**				
≤8	0.170 (0.098-0.295)	<0.001*	0.186 (0.104-0.333)	<0.001*
>8				
**Radiation**				
No	0.571 (0.397-0.821)	0.002*	0.668 (0.451-0.988)	0.043*
Yes				
**LDH level**				
≤ULN	1.471 (1.026-2.111)	0.036*	1.339 (0.927-1.934)	0.120
>ULN				
**Group**				
irAE(-)	0.420 (0.301-0.585)	<0.001*	0.565 (0.397-0.806)	0.002*
irAE(+)				

ECOG PS, Eastern Cooperative Oncology Group Performance Status; HR, hazard ratio; CI, confidence interval; LDH, lactate dehydrogenase; ULN, upper limit of normal; irAEs, immune-related adverse events; OS, overall survival. *p<0.05.

Age, sex, history of surgery, history of smoking, and history of alcohol consumption were not associated with PFS or OS (**Supplementary Table 2**).

### Prognostic factors predicting irAEs

Patients who received PD-1 inhibitors for > 8 cycles, combination radiation, or antiangiogenic therapy during immunotherapy were found to have an increased risk of irAEs by univariate and multivariate analyses. No significant associations between irAEs and age, sex, ECOG PS, therapy line, or a number of organs with metastases and LDH level were observed (**Table 5**).

**Table 5:** Results of univariate and multivariate analyses showing factors affecting the irAEs.

Variables	Univariate analysis		Multivariate analysis	
	OR (95% CI)	p value	OR (95% CI)	p value
Age (>60)	1.443 (0.913-2.283)	0.117		
Sex (female)	1.217 (0.605-2.446)	0.582		
ECOG PS (≥ 2)	0.658 (0.410-1.056)	0.083		
Therapy line (≥ 2)	0.681 (0.410-1.130)	0.137		
Number of organs with metastases (≥ 2)	0.741 (0.457-1.201)	0.224		
Cycles>8	3.109 (1.776-5.444)	<0.001*	3.062 (1.726-5.432)	<0.001*
Radiation	2.121 (1.288-3.492)	0.003*	2.332 (1.382-3.936)	0.002*
Antiangiogenic therapy	1.934 (1.007-3.711)	0.047*	2.189 (1.101-4.353)	0.025*
LDH level (>ULN)	0.763 (0.440-1.325)	0.337		

ECOG PS, Eastern Cooperative Oncology Group Performance Status; OR, odds ratio; CI, confidence interval; LDH, lactate dehydrogenase; ULN, upper limit of normal. *p<0.05.

## Discussion

ICIs have shown superior therapeutic efficacy and prognosis in patients with EC. However, ICIs treatment is frequently accompanied by irAEs. It is unclear whether the development of irAEs is related to the better outcome of ICIs in patients with EC. To our knowledge, this study demonstrates that patients with irAEs had superior outcomes from PD-1 inhibitors, including higher ORR and DCR, and better PFS and OS in patients with advanced EC.

A significant association has been reported with increased survival after ICIs treatment for irAEs in gastrointestinal cancer (20) or NSCLC (16, 17). However, whether the occurrence of irAEs indicates a superior response and survival outcomes in melanoma patients remains contentious (21, 22). This indicates that the differences in the association between irAEs and ICIs treatment may differ in different tumors. Thus, this study evaluated the association between irAEs and the clinical outcomes of PD-1 inhibitors treated in advanced EC. Although the actual pathophysiology of irAEs has not been completely elucidated, various mechanisms have been reported to explain the development of irAEs. IrAEs may be triggered by antigens commonly shared by tumors and normal tissues, which then release T cells to attack these two tissues, producing both response and toxicity. In a prospective study of 73 NSCLC patients who received PD-1 inhibitors, TCR clonotype analysis was made on four patients with skin irAEs, and common T-cell clones were found to exist in both the skin and tumor in four patients (23). Another study showed that pre-existing organ-specific antigen exposure may be responsible for the irAEs from ICIs (4, 24).

The potential risk factors, along with supporting evidence, include potential germline genetic factors, autoimmune diseases, radiotherapy, chemotherapy, targeted therapy, and preexisting autoantibodies (25, 26). In the present study, combination treatment with ICIs and radiation or antiangiogenic therapy were risk factors for irAEs.

Mounting evidence indicates that concomitant use of chemotherapy, targeted therapy, or radiotherapy with ICIs enhances efficacy, but leads to the risk of augmented treatment toxicities (26–28). In addition to inducing immunogenic cell death and priming and activation of naive T cells (29), radiotherapy can also produce immunogenic damage to nontumor cells and increase immune cell infiltration, leading to increased irAEs when combined with ICIs. Antiangiogenic therapies can induce the upregulation of PD-L1 in endothelial and tumor cells, resulting in an increased risk of irAEs when combined with ICIs (30). In contrast, the finding of > 8 PD-1 inhibitors > 8 cycles was also related to the high risk of irAEs in this study. Prolonged ICIs administration may result in a higher incidence of irAEs. With the increase in ICIs treatment cycles and doses, the enhanced activity of T lymphocytes and high levels of cytokines and inflammatory factors lead to side effects in normal tissues (31). Multi-institutional randomized controlled trials are required to identify predictive biomarkers of irAEs.

Several retrospective studies found that patients with multiple irAEs have better survival outcomes than in those with single or no irAEs (16, 32, 33), which may be explained by the development of multiple irAEs reflecting the immune system effectively targeting several organs and sustaining antitumor responses. However, those studies were mostly restricted to a handful of patients included, and the mechanisms of this association have yet to be identified. Interestingly, our study showed that patients with a single irAE had longer PFS and OS than those with multiple irAEs and no irAEs. In total, 29 (9.83%) patients had ≥ 3 grade irAEs among patients with multiple irAEs. The result of patients with multiple irAEs having a worse prognosis than those with single irAEs may be attributed to the serious adverse events that improve the danger of death and neutralize the efficacy of ICIs. Thus, additional studies are needed to elucidate the association between irAEs and ICIs efficacy (34).

This study has several limitations. Most importantly, this was a retrospective investigation, and there was an unavoidable bias in the selection of patients and potential confounding factors. Second, the mechanisms of irAEs were unclear; thus, further studies are required to illustrate the related results. Considering these limitations, we should carefully interpret the current results and conduct prospective studies to verify the findings of the association between irAEs and ICIs efficacy.

## Conclusion

The occurrence of irAEs predicts better survival outcomes, including patients receiving PD-1 inhibitors ≤ 8 cycles, in advanced EC. We believe that the development of irAEs can potentially be an effective and promising marker of survival in advanced EC.

## Data availability statement

The original contributions presented in the study are included in the article/**Supplementary Material**. Further inquiries can be directed to the corresponding author.

## Ethics statement

This study was reviewed and approved by institutional review board of the Shandong Cancer Hospital and Institute. Written informed consent was obtained from all participants for their participation in this study.

## Author contributions

Data collection, WQ and YD. Manuscript preparation, data analysis, WQ, LY, BF and BZ. Visualization, YD, BL and LW. Project administration, LW. Funding acquisition, LW. All authors contributed to the article and approved the submitted version.

## Funding

This research was supported by Natural Science Foundation of Shandong Province, (Grant number ZR2019LZL012), National Natural Science Foundation of China (Grant number 8217102892), Jinan Clinical Medical Science and Technology Innovation Plan (Grant number 202019043), The Key Research and Development Program of Shandong (Major Science & Technology Innovation Project) (2021SFGC0501) and Start-up fund of Shandong Cancer Hospital (2020-B14).

## Conflict of interest

The authors declare that the research was conducted in the absence of any commercial or financial relationships that could be construed as a potential conflict of interest.

## Publisher’s note

All claims expressed in this article are solely those of the authors and do not necessarily represent those of their affiliated organizations, or those of the publisher, the editors and the reviewers. Any product that may be evaluated in this article, or claim that may be made by its manufacturer, is not guaranteed or endorsed by the publisher.

## References

[1] Bray F Ferlay J Soerjomataram I Siegel RL Torre LA Jemal A . Global cancer statistics 2018: GLOBOCAN estimates of incidence and mortality worldwide for 36 cancers in 185 countries. CA Cancer J Clin (2018) 68(6):394–424. doi: 10.3322/caac.21492 30207593

[2] Abdo J Agrawal DK Mittal SK . Basis for molecular diagnostics and immunotherapy for esophageal cancer. Expert Rev Anticancer Ther (2017) 17(1):33–45. doi: 10.1080/14737140.2017.1260449 27838937 PMC5542819

[3] Hong Y Ding ZY . PD-1 inhibitors in the advanced esophageal cancer. Front Pharmacol (2019) 10:1418. doi: 10.3389/fphar.2019.01418 31920637 PMC6916418

[4] Das S Johnson DB . Immune-related adverse events and anti-tumor efficacy of immune checkpoint inhibitors. J immunother Cancer (2019) 7(1):306. doi: 10.1186/s40425-019-0805-8 31730012 PMC6858629

[5] Doki Y Ajani JA Kato K Xu J Wyrwicz L Motoyama S . Nivolumab combination therapy in advanced esophageal squamous-cell carcinoma. New Engl J Med (2022) 386(5):449–62. doi: 10.1056/NEJMoa2111380 35108470

[6] Janjigian YY Shitara K Moehler M Garrido M Salman P Shen L . First-line nivolumab plus chemotherapy versus chemotherapy alone for advanced gastric, gastro-oesophageal junction, and oesophageal adenocarcinoma (CheckMate 649): A randomised, open-label, phase 3 trial. Lancet (London England) (2021) 398(10294):27–40. doi: 10.1016/s0140-6736(21)00797-2 PMC8436782 34102137

[7] Sun JM Shen L Shah MA Enzinger P Adenis A Doi T . Pembrolizumab plus chemotherapy versus chemotherapy alone for first-line treatment of advanced oesophageal cancer (KEYNOTE-590): A randomised, placebo-controlled, phase 3 study. Lancet (London England) (2021) 398(10302):759–71. doi: 10.1016/s0140-6736(21)01234-4 34454674

[8] Luo H Lu J Bai Y Mao T Wang J Fan Q . Effect of camrelizumab vs placebo added to chemotherapy on survival and progression-free survival in patients with advanced or metastatic esophageal squamous cell carcinoma: The ESCORT-1st randomized clinical trial. Jama (2021) 326(10):916–25. doi: 10.1001/jama.2021.12836 PMC8441593 34519801

[9] Fujii T Colen RR Bilen MA Hess KR Hajjar J Suarez-Almazor ME . Incidence of immune-related adverse events and its association with treatment outcomes: the MD Anderson cancer center experience. Investigat New Drugs (2018) 36(4):638–46. doi: 10.1007/s10637-017-0534-0 PMC5962379 29159766

[10] Kennedy LB Salama AKS . Salama AKS. A review of cancer immunotherapy toxicity (2020) 70(2):86–104. doi: 10.3322/caac.21596 31944278

[11] Arnaud-Coffin P Maillet D Gan HK Stelmes JJ You B Dalle S . A systematic review of adverse events in randomized trials assessing immune checkpoint inhibitors. Int J Cancer (2019) 145(3):639–48. doi: 10.1002/ijc.32132 30653255

[12] Henderson Berg MH Del Rincón SV Miller WH . Potential therapies for immune-related adverse events associated with immune checkpoint inhibition: from monoclonal antibodies to kinase inhibition. J immunother Cancer (2022) 10(1). doi: 10.1136/jitc-2021-003551 PMC8796266 35086945

[13] Spiers L Coupe N Payne M . Toxicities associated with checkpoint inhibitors-an overview. Rheumatol (Oxford England) (2019) 58(Suppl 7):vii7–vii16. doi: 10.1093/rheumatology/kez418 PMC6900917 31816085

[14] Khoja L Day D Wei-Wu Chen T Siu LL Hansen AR . Tumour- and class-specific patterns of immune-related adverse events of immune checkpoint inhibitors: a systematic review. Ann Oncol (2017) 28(10):2377–85. doi: 10.1093/annonc/mdx286 28945858

[15] Luke JJ Lemons JM Karrison TG Pitroda SP Melotek JM Zha Y . Safety and clinical activity of pembrolizumab and multisite stereotactic body radiotherapy in patients with advanced solid tumors. J Clin Oncol (2018) 36(16):1611–8. doi: 10.1200/jco.2017.76.2229 PMC5978468 29437535

[16] Ricciuti B Genova C De Giglio A Bassanelli M Dal Bello MG Metro G . Impact of immune-related adverse events on survival in patients with advanced non-small cell lung cancer treated with nivolumab: long-term outcomes from a multi-institutional analysis. J Cancer Res Clin Oncol (2019) 145(2):479–85. doi: 10.1007/s00432-018-2805-3 PMC11810236 30506406

[17] Haratani K Hayashi H Chiba Y Kudo K Yonesaka K Kato R . Association of immune-related adverse events with nivolumab efficacy in non-Small-Cell lung cancer. JAMA Oncol (2018) 4(3):374–8. doi: 10.1001/jamaoncol.2017.2925 PMC6583041 28975219

[18] Ishihara H Takagi T Kondo T Homma C Tachibana H Fukuda H. . Association between immune-related adverse events and prognosis in patients with metastatic renal cell carcinoma treated with nivolumab. Urologic Oncol (2019) 37(6):355.e21–.e29. doi: 10.1016/j.urolonc.2019.03.003 30935847

[19] Sato K Akamatsu H Murakami E Sasaki S Kanai K Hayata A . Correlation between immune-related adverse events and efficacy in non-small cell lung cancer treated with nivolumab. Lung Cancer (2018) 115:71–4. doi: 10.1016/j.lungcan.2017.11.019 29290265

[20] Masuda K Shoji H Nagashima K Yamamoto S Ishikawa M Imazeki H . Correlation between immune-related adverse events and prognosis in patients with gastric cancer treated with nivolumab. BMC Cancer (2019) 19(1):974. doi: 10.1186/s12885-019-6150-y 31638948 PMC6805586

[21] Weber JS Hodi FS Wolchok JD Topalian SL Schadendorf D Larkin J . Safety profile of nivolumab monotherapy: A pooled analysis of patients with advanced melanoma. J Clin Oncol (2017) 35(7):785–92. doi: 10.1200/jco.2015.66.1389 28068177

[22] Indini A Di Guardo L Cimminiello C Prisciandaro M Randon G De Braud F . Immune-related adverse events correlate with improved survival in patients undergoing anti-PD1 immunotherapy for metastatic melanoma. J Cancer Res Clin Oncol (2019) 145(2):511–21. doi: 10.1007/s00432-018-2819-x PMC11810247 30539281

[23] Berner F Bomze D Diem S Ali OH Fässler M Ring S . Association of checkpoint inhibitor-induced toxic effects with shared cancer and tissue antigens in non-small cell lung cancer. JAMA Oncol (2019) 5(7):1043–7. doi: 10.1001/jamaoncol.2019.0402 PMC6487908 31021392

[24] Iwama S De Remigis A Callahan MK Slovin SF Wolchok JD Caturegli P . Pituitary expression of CTLA-4 mediates hypophysitis secondary to administration of CTLA-4 blocking antibody. Sci Trans Med (2014) 6(230):230ra45. doi: 10.1126/scitranslmed.3008002 24695685

[25] Bagchi S Yuan R Engleman EG . Immune checkpoint inhibitors for the treatment of cancer: Clinical impact and mechanisms of response and resistance. Annu Rev Pathol (2021) 16:223–49. doi: 10.1146/annurev-pathol-042020-042741 33197221

[26] Liu X Shi Y Zhang D Zhou Q Liu J Chen M . Risk factors for immune-related adverse events: what have we learned and what lies ahead? biomark Res (2021) 9(1):79. doi: 10.1186/s40364-021-00314-8 34732257 PMC8565046

[27] Ramos-Casals M Brahmer JR Callahan MK Flores-Chávez A Keegan N Khamashta MA . Immune-related adverse events of checkpoint inhibitors. Nat Rev Dis Primers (2020) 6(1):38. doi: 10.1038/s41572-020-0160-6 32382051 PMC9728094

[28] Zhou X Yao Z Bai H Duan J Wang Z Wang X . Treatment-related adverse events of PD-1 and PD-L1 inhibitor-based combination therapies in clinical trials: a systematic review and meta-analysis. Lancet Oncol (2021) 22(9):1265–74. doi: 10.1016/s1470-2045(21)00333-8 34391508

[29] Twyman-Saint Victor C Rech AJ Maity A Rengan R Pauken KE Stelekati E . Radiation and dual checkpoint blockade activate non-redundant immune mechanisms in cancer. Nature (2015) 520(7547):373–7. doi: 10.1038/nature14292 PMC4401634 25754329

[30] Gao L Yang X Yi C Zhu H . Adverse events of concurrent immune checkpoint inhibitors and antiangiogenic agents: A systematic review. Front Pharmacol (2019) 10:1173. doi: 10.3389/fphar.2019.01173 31680957 PMC6812341

[31] Maker AV Yang JC Sherry RM Topalian SL Kammula US Royal RE . Intrapatient dose escalation of anti-CTLA-4 antibody in patients with metastatic melanoma. J immunother (Hagerstown Md: 1997) (2006) 29(4):455–63. doi: 10.1097/01.cji.0000208259.73167.58 PMC2134804 16799341

[32] Shimozaki K Sukawa Y Beppu N Kurihara I Suzuki S Mizuno R . Multiple immune-related adverse events and anti-tumor efficacy: Real-world data on various solid tumors. Cancer Manag Res (2020) 12:4585–93. doi: 10.2147/cmar.S247554 PMC7305832 32606951

[33] Paderi A Giorgione R Giommoni E Mela MM Rossi V Doni L . Association between immune related adverse events and outcome in patients with metastatic renal cell carcinoma treated with immune checkpoint inhibitors. Cancers (2021) 13(4):860. doi: 10.3390/cancers13040860 33670634 PMC7922597

[34] Zhong L Wu Q Chen F Liu J Xie X . Immune-related adverse events: promising predictors for efficacy of immune checkpoint inhibitors. Cancer immunol immunother (2021) 70(9):2559–76. doi: 10.1007/s00262-020-02803-5 PMC10991616 33576872

