# Supplementary material for: Association between immune-related adverse events and the efficacy of PD-1 inhibitors in advanced esophageal cancer
Source: Front Immunol. 2022 Sep 28;13:931429. doi: 10.3389/fimmu.2022.931429 (PMC9554876; doi:10.3389/fimmu.2022.931429)
Supplement: Supplementary file 1 [file Table_1.docx]

Supplementary Material

**Supplementary TABLE 1 |** Baseline characteristics of patients

| characteristic | All patients  N(%) | IrAE - group  N(%) | IrAE + group  N(%) | p value |
| --- | --- | --- | --- | --- |
| Total N | 295 | 152(51.53) | 143(48.47) |  |
| **Age（years）** |  |  |  |  |
| Median(range) | 60(36-84) | 60(43-84) | 61(36-76) | 0.201 |
| **Gender** |  |  |  |  |
| Male | 259(87.8) | 135(88.8) | 124(86.7) | 0.581 |
| Female | 36(12.2) | 17(11.2) | 19(13.3) |  |
| **ECOG PS** |  |  |  |  |
| ＜2 | 181(61.4) | 86(56.6) | 95(66.4) | 0.082 |
| ≥2 | 114(38.6) | 66(43.4) | 48(33.6) |  |
| **Tumor location** |  |  |  |  |
| Cervical esophagus/ Upper thoracic | 43(14.6) | 22(14.5) | 21(14.7) | 0.691 |
| Middle thoracic | 118(40.0) | 65(42.8) | 53(37.1) |  |
| Lower thoracic | 110(37.3) | 52(34.2) | 58(40.6) |  |
| Other | 24(8.1) | 13(8.6) | 11(7.7) |  |
| **History of surgery** |  |  |  |  |
| Yes | 123(41.7) | 59(38.8) | 64(44.8) | 0.301 |
| No | 172(58.3) | 93(61.2) | 79(55.2) |  |
| **Histology** |  |  |  |  |
| Squamous cell carcinoma | 278(94.2) | 145(95.4) | 133(93.0) | 0.465 |
| Adenocarcinoma | 7(2.4) | 2(1.3) | 5(3.5) |  |
| Other | 10(3.4) | 5(3.3) | 5(3.5) |  |
| **Number of organs with metastases** |  |  |  |  |
| <2 | 194(65.8) | 95(62.5) | 99(69.2) | 0.223 |
| ≥2 | 10134.2) | 57(37.5) | 44(30.8) |  |
| **Site of metastases** |  |  |  |  |
| Lung | 62(21.0) | 31(20.4) | 31(21.7) | 0.787 |
| Liver | 67(22.7) | 39(25.7) | 28(19.6) | 0.213 |
| Bone | 36(12.2) | 19(12.5) | 17(11.9) | 0.873 |
| Brain | 6(2.0) | 2(1.3) | 4(2.8) | 0.368 |
| **Immunotherapy line** |  |  |  |  |
| 1st | 85(28.8) | 38(25.0) | 47(32.9) | 0.136 |
| ≥2nd | 210(71.2) | 114(75.0) | 96(67.1) |  |
| **History of drinking** |  |  |  |  |
| Yes | 150(50.8) | 81(53.3) | 69(48.3) | 0.387 |
| No | 145(49.2) | 71(46.7) | 74(51.7) |  |
| **History of smoking** |  |  |  |  |
| Yes | 157(53.2) | 84(55.3) | 73(51.0) | 0.468 |
| No | 138(46.8) | 68(44.7) | 70(49.0) |  |
| **LDH level** |  |  |  |  |
| ≤ULN | 224(75.9) | 112(73.7) | 112(78.3) | 0.352 |
| >ULN | 71(24.1) | 40(26.3) | 31(21.7) |  |
| **Stage** |  |  |  |  |
| II | 6(2.0) | 2(1.3) | 4(2.8) | 0.496 |
| III | 41(13.9) | 19(12.5) | 22(15.4) |  |
| IV | 248(84.1) | 131(86.2) | 117(81.8) |  |

irAE, immune-related adverse event; ECOG PS, Eastern Cooperative Oncology Group Performance Status; LDH, lactate dehydrogenase; ULN, upper limit of normal

**Supplementary TABLE 2 |** Results of univariate analyses showing factors affecting progression-free survival and overall survival.

| Variables | Univariate analysis | | | |
| --- | --- | --- | --- | --- |
|  | HR for PFS (95% CI) | p value | HR for OS (95% CI) | p value |
| **Age（years）** |  |  |  |  |
| <60 | 0.744 (0.549-1.007) | 0.056 | 1.180 (0.861-1.616) | 0.303 |
| ≥60 |  |  |  |  |
| **Gender** |  |  |  |  |
| Male | 1.074 (.0678-1.702) | 0.761 | 0.904 (0.565-1.447) | 0.904 |
| Female |  |  |  |  |
| **History of surgery** |  |  |  |  |
| Yes | 1.072 (0.793-1.449) | 0.653 | 0.939 (0.684-1.289) | 0.697 |
| No |  |  |  |  |
| **History of drinking** |  |  |  |  |
| Yes | 1.107 (0.820-1.4950 | 0.507 | 1.073 (0.782-1.470) | 0.663 |
| No |  |  |  |  |
| **History of smoking** |  |  |  |  |
| Yes | 0.954 (0.706-1.288) | 0.758 | 1.047 (0.763-1.437) | 0.774 |
| No |  |  |  |  |
